# Temporal niche differentiation often leads to priority effects rather than coexistence: Lessons from a marine midge

**DOI:** 10.1111/1365-2656.70094

**Published:** 2025-07-20

**Authors:** Runa K. Ekrem, Charlotte de Vries, Tobias S. Kaiser, Hanna Kokko

**Affiliations:** ^1^ Department of Evolutionary Biology and Environmental Studies University of Zurich Zurich Switzerland; ^2^ Institute for Environmental Sciences, Leiden University Leiden The Netherlands; ^3^ Theoretical Biology & Bioinformatics, University of Utrecht Utrecht The Netherlands; ^4^ Max Planck Research Group Biological Clocks Max Planck Institute for Evolutionary Biology Plön Germany; ^5^ Institute of Organismic and Molecular Evolution (iomE) University of Mainz Mainz Germany; ^6^ Institute for Quantitative and Computational Biosciences University of Mainz Mainz Germany

**Keywords:** coexistence, competitive exclusion, mate‐finding Allee effect, polymorphism, priority effect, temporal niche

## Abstract

1. While niche differences aid coexistence, the role of temporal niches is complex. A recent study (Stump & Vasseur, 2023) casts doubt on the idea that species coexist easily if they partition abiotic niches that vary in time. The storage effect, which aids coexistence, requires that species differ in what is a ‘good year’, and that the benefits that the currently common species can draw from its own good year become limited due to intraspecific competition.
2. The recent re‐evaluation of temporal niches considered Allee effects only fleetingly. We complement their work by providing a case study of the marine midge *Clunio marinus*, where coexistence appears to occur in nature, is associated with a strong difference in timing traits, and also features Allee effects because rare timing phenotypes emerge with limited mating opportunities.
3. The larvae develop in the sea, and adults emerge and mate during the lowest low tides. These tides coincide with either the full or the new moon, and genetically determined strains use either one of them, or both, for emergence. A ‘good year’ in this system translates into a particular low tide. Allee effects create strain‐specific good tides if the risk of hybridization is greater for the currently rare strain, which mates more often with another strain, than the currently common strain.
4. We are able to investigate this effect by varying the effects of hybridization in our model of *Clunio* biology. Temporal niches, mate‐finding Allee effects, hybridization possibilities and a potential growth‐survival tradeoff do not easily combine to yield stable coexistence. Most factors instead promote positive frequency dependence, leading to priority effects. Ontogenetic niche shifts among larvae deviate from this result: if suitably timed, they are able to concentrate competition in a coexistence‐promoting manner.
5. Our study thus complements and strengthens Stump and Vasseur's conclusion that a finding of temporal niche differentiation should not be straightforwardly assumed to be an explanation for the coexistence of two or more morphs or species. We encourage linking temporal niche studies with those of priority effects, as well as the study of other coexistence mechanisms that may operate within systems that feature temporal niches.

## Graphical abstract

When organisms use similar resources but at a different time, it is not obvious whether they can divide their temporal niches in a coexistence promoting manner or of something else should happen. This study shows that priority effects are a likely, but not self‐evident, outcome.

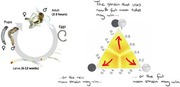

## 1 INTRODUCTION

The competitive exclusion principle (also called Gause's law) is one of the fundamental tenets of ecology. It states that stable coexistence is impossible if two species compete for the exact same limited resource (Gause, 1934; Hardin, 1960). Whenever competitors coexist, the quest therefore is to understand what part of the resource spectrum might be used differently by the two species or strains (Polechová & Storch, 2008; see also Spaak & De Laender, 2020 for an up‐to‐date view on the mathematical basis of niche definitions), or whether there are other ways to promote coexistence, for example, stochasticity combining with dispersal (Gravel et al., 2006; Bode et al., 2011; Pigot et al., 2018).

An interesting aspect of niches is that competitors may divide resources not only in terms of ‘what’ or ‘where’, but also ‘when’ (Gao et al., 2020). In two competing frog species, for example, shifts in phenology have strong effects on interspecific competition between tadpoles, with consequences for survival and biomass (Rudolf, 2018). Differences in timing of activities (allochrony) are often argued to aid coexistence (Albrecht & Gotelli, 2001; Arlettaz et al., 2017; Gao et al., 2020; Menges et al., 2024; Robertson, 1895; Waser, 2015) and even contribute to speciation (Bouaouina et al., 2023; Taylor & Friesen, 2017).

At first sight, allochrony appears to be a fully equivalent alternative to microhabitat differences in the spatial realm, both being able to separate species into distinct niches. They are, however, not interchangeable. A population that specializes to use one habitat to an extent that it fails in others can in principle persist, while it is not possible to completely neglect the need to persist (at minimum, using dormant states) through all points along a temporal axis. Work on temporal niches has therefore focused on the so‐called ‘storage effect’ (Barabás et al., 2018; Chesson, 2000), where competitors specialize in growing at different environmental conditions that fluctuate over time while also persisting over the (species‐specific) poor season (Johnson & Hastings, 2022; Kelly & Bowler, 2005; Tan et al., 2013). However, as pointed out already by Case and Gilpin (1974), using the same resource at different times does not necessarily remove competition: instead, species A may deplete the resource from the perspective of species B that arrives at the resource later. Thus, A may simply outcompete B via a priority effect (Zou & Rudolf, 2023).

Recently, Stump and Vasseur (2023) provided a thorough explanation of theory as well as a re‐examination of three real‐world systems where temporal niche differentiation has been argued to aid coexistence. They caution against the temptation to interpret timing differences too straightforwardly as evidence that the storage effect is operating and that coexistence is thus explained. Similarly, recent work by van Doorn et al. (2025) on a specific system (a moth *Spodoptera frugiperda*) showed that temporal differentiation of two strains does not lead to stable divergence. Extra ingredients, in their case intralocus sexual conflict, may be needed before reproductive isolation is maintained, which warns against too straightforward assumptions that allochrony automatically explains coexistence. Here we complement and extend these recent evaluations by exploring coexistence and temporal niches in a marine midge, *Clunio marinus*.

*Clunio marinus* only feeds during the benthic larval stage. Adult lifespan (a few hours) is temporally restricted to the lowest low tides, during which males hover above the water surface searching for virgin females. Eggs are laid among algae exposed by the low tide and are soon covered by the incoming tide (Kaiser et al., 2021). Larval development times vary between 6 and 14 weeks in the laboratory (Neumann, 1966), with potential for faster development in the field. The emergence of a cohort is thus spread over ca. 2 months, giving each larva several emergence options. Emergence times are genetically determined and associate with either the new or the full moon (circalunar rhythm, i.e. monthly emergence), or both (circasemilunar rhythm, i.e. fortnightly emergence) (Kaiser et al., 2021; Neumann, 2014). Sites vary in the identity of co‐occurring strains (Kaiser et al., 2021).

*C. marinus* offers an interesting way to extend the work of Stump and Vasseur (2023). These authors considered Allee effects fleetingly in one specific context (when evaluating the success of models that assume infinite population sizes), while in *C. marinus*, Allee effects due to mating interactions are central in creating strain‐specific ‘good’ and ‘bad’ times of emergence. Hybrid production is not equally distributed across strains that vary in abundance. Unless the number of adults of two (or more) strains emerging at a specific tide are precisely equal, a currently rare strain experiences far more mate encounters that can lead to hybridization than a currently common strain (Irwin & Schluter, 2022; Kyogoku & Kokko, 2020). Hybridization in this system yields maladaptive timing traits: hybrids emerge when the tides are not low enough to expose sufficient habitat, and/or fail to emerge in synchrony with potential mating partners (Kaiser et al., 2011). Given that hybridization is unlikely to be beneficial, strains effectively create their own ‘good tides’—those when most co‐emerging individuals are of the same strain. Tides, here, are analogous to ‘good years’ in the review of Stump and Vasseur (2023). They point out that for a temporal storage effect to promote coexistence, the benefits that a particular species enjoys from its own good year should be limited due to the concomitant increase in intraspecific competition.

Evaluating our *Clunio* case against this theoretical background requires formal modelling. Strains effectively create their own ‘good tides’ via Allee effects and hybridization, but whether the consequently elevated breeding success yields increased intra‐strain competition (important according to Stump & Vasseur, 2023) is not a priori easy to assess. While high reproductive success places larvae into the same areas simultaneously, larval developmental times are much longer than the 2‐week interval between the most suitable tides for reproduction (full and new moon), and thus the same sites will merge larval cohorts from different tides. Whether all larvae compete for identical resources, or shift as they age, could also be important: ontogenetic niche shifts can impact coexistence (Anaya‐Rojas et al., 2023; Miller & Rudolf, 2011; Schellekens et al., 2010). Finally, as one of the strains uses both full and new moon opportunities, its time in the water is on average shorter than that of individuals that emerge only at one of those moon phases. This creates potential asymmetries between the strains: longer time spent in the water implies more time for growth but also a larger number of days during which the larva may succumb to predation (growth‐survival tradeoff).

To sum up, our model investigates various hybridization scenarios, in the absence or presence of Allee effects, growth‐survival trade‐offs or ontogenetic niche shifts. While the list of factors considered is large, it is important to do an exhaustive search of the mechanisms that may promote or hinder the ability of temporal niches to support coexistence, as we aim to provide an overall assessment for our system's ability to do so.

## 2 MATERIALS AND METHODS

### 2.1 Biological background

*Clunio* possesses features that necessitate a tailor‐made model. First, the coexistence question does not refer to species, but to genetically determined strains (Kaiser et al., 2021). Second, the species is a capital breeder with short‐lived, non‐feeding adults. All growth happens before emergence, but because it is impossible to age larvae in the wild, we lack information for the magnitude of the size improvement (if any) that associates with a longer period of growth. We thus vary the growth‐survival tradeoff from absent to very strong.

We deal with a third factor, ontogenetic niche shifts, similarly: as we lack detailed knowledge of the resource use of different‐sized larvae, we simply contrast two assumption sets: a *single niche* scenario where all larvae feed on the same resource, and an *ontogenetic niche shift* (henceforth *niche shift*) scenario where larvae use niches age‐dependently.

The final complicating factor is that the extremely short lifespan of adults limits the time window during which a mate must be found, potentially leading to a mate‐finding Allee effect (Fauvergue, 2013; Gascoigne et al., 2009). The mating success of an already common strain may thus be improved, making coexistence harder; and if rare strains co‐occur in their temporal mating niche with a common one, maladaptive hybridization risks materialize more often for the rare strain, again harming coexistence.

### 2.2 Model with three time scales

The model operates over three time scales: (i) from one season to the next (yielding the potential to consider multiple years); (ii) within each season, there are multiple weekly events (relevant for larval life history, including hatching, surviving to the next week, maturing to enter a stage that waits for the next tide suitable for emergence); and (iii) mating, which occurs during one suitable low tide. All weekly events are applied as simultaneous transitions between discrete states that describe the classes of individuals. The mating of adults, for which there is only a short time window available, is modelled in terms of hours and in continuous time (Kokko, 2024).

### 2.3 Strains and genetic architecture

The exact genetic architecture of timing traits in *C. marinus* is unknown (Kaiser et al., 2016, 2021; Kaiser & Heckel, 2012). For conceptual simplicity, we assume a single locus with three alleles creating all timing phenotypes when homozygous. E_M1_ and E_M2_ (E for ‘emergence’ and M for ‘monthly’) emerge once every 4 weeks, with their periods shifted by 2 weeks with respect to each other. We assume that *E*_*M*1_ emerges if the week number is divisible by 4, and *E*_*M*2_ emerges if (week number + 2) is divisible by 4. In single‐season simulations, we arbitrarily assign *E*_*M*1_ to start first. In multi‐year simulations, we additionally need to take into account that either may emerge first (experience a head start) in any given year: weather in the spring starts to permit emergence independently of phases of the moon, making the identity of the first emerging strain effectively a coin flip. The third, fortnightly strain (named *E*_F_) emerges every 2 weeks and uses every low tide that is used by either *E*_*M*1_ or *E*_*M*2_.

*E*_F_ may co‐emerge with either *E*_*M*1_ or *E*_*M*2_, and the resulting hybrids possess intermediate timing phenotypes that are ecologically suboptimal with respect to tides (Kaiser et al., 2011, 2021). For conceptual clarity we implement hybrid maladaptation with reduced viability (a constant factor *γ* < 1 relative to viability of pure‐strain eggs; see Table S1 for notation). As it is difficult to ascertain the dominance structure of the alleles, we ran all models assuming full dominance, where *E*_F_ is recessive to the *E*_*M*1_ and *E*_*M*2_, as well as the opposite assumption structure, where *E*_F_ is dominant (we do not show the latter assumption set, as it yields no qualitative differences to the outcomes we report below; also note that coexistence‐promoting dominance structures, such as heterozygote advantage, are unlikely in a setting where hybrids have poorly chosen timing traits). Under our simplifying assumptions, *E*_*M*1_ *E*_*M*2_ hybrids are never produced since the temporal mating niches of *E*_*M*1_ and *E*_*M*2_ do not overlap.

### 2.4 Life cycle

We assume each season consists of 44 weeks (a year minus a few weeks of winter, during which no mating occurs). To pick a random moon phase for the first week, we consider a season to consist of Weeks 1–44, 2–45, 3–46 or 4–47, all occurring with equal probability (this creates proper moon phases in the modulo computations below). A season can fit in multiple life cycles, but the exact number of generations in one season is not pre‐specified, as larval development times vary. In multi‐year versions of the model, we collect the eggs at the end of the season to form the egg pool that begins each new season in the next year.

**FIGURE 1:**
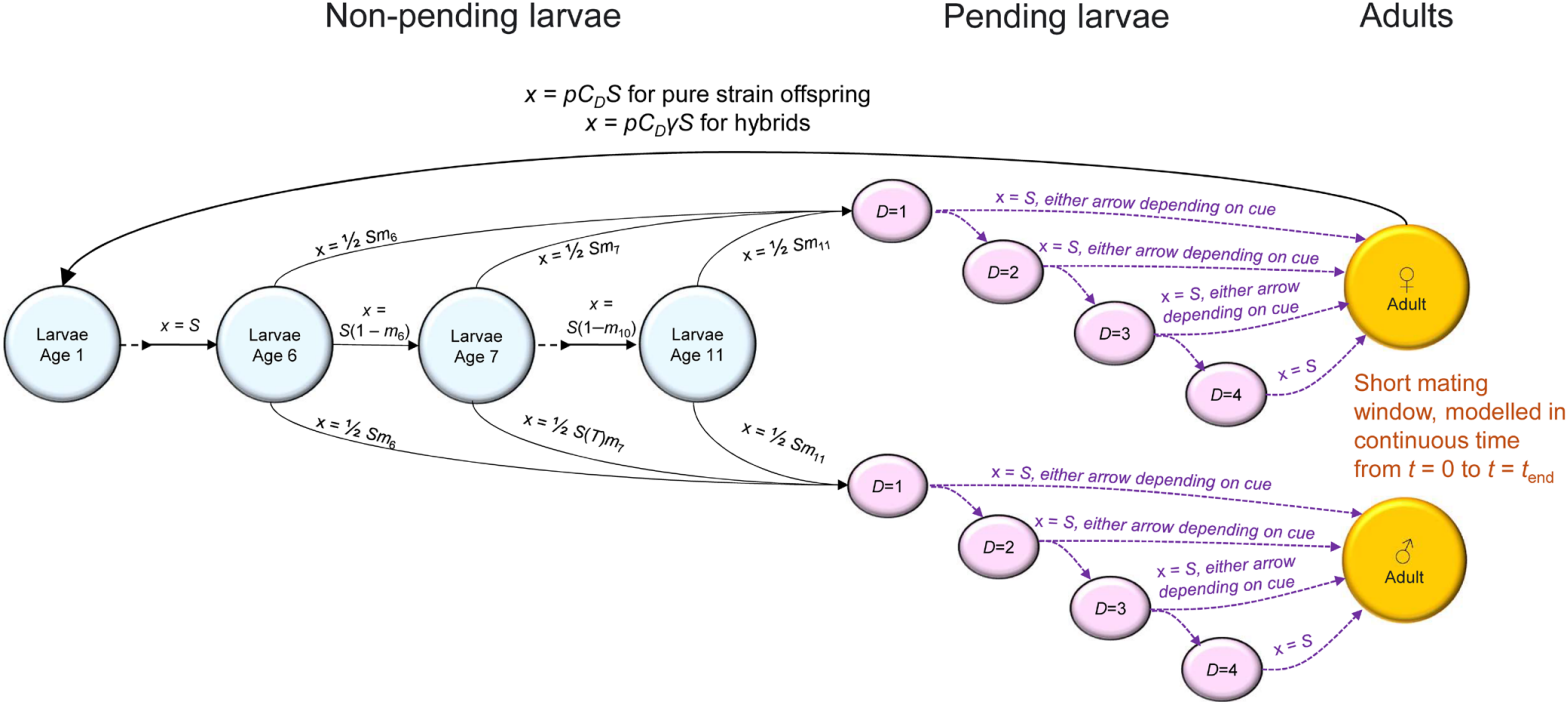
Overview of the within‐season dynamics with circles representing life stages, and each arrow associating with a transition, the value of which (*x*) is given alongside the arrow; for brevity we write survival as *S* rather than *S*(*T*) (see text for complete notation). If adults (orange) exist, they mate within the mating dynamics timescale; all other transitions (arrows) take 1 week to complete and occur simultaneously with respect to each other. From age of 6 weeks onwards, it is possible for a larva (blue) to transition to a pending state (pink), and from this point onwards, emergence to become an adult happens if the cue is right (long purple arrows), the alternative being continued existence in the water (short purple arrows). Note that no individual has to wait longer than 4 weeks for the appropriate cue, and that we track the number of weeks spent in the pending state, *D*, for males too, but this has any impact on performance as an adult for females as an optional increase of fecundity with *D*.

Each week has multiple simultaneous transitions between stages (Figure 1): the youngest age class enters the population of larvae, other larvae age by 1 week, sufficiently developed larvae enter a *pending* state, which we define as a state where a suitable cue (full or new moon) will trigger emergence, and emergence occurs if there are pending larvae that receive a suitable cue. Each transition takes the general form

(1) $$N_{\to \text{state}}\left(T\right)={\mathrm{xN}}_{\text{state}\to }\left(T\right),$$

where _→state_ denotes the incoming state (the state of individuals after the transition), _state→_ denotes the state that the transitioning happens from, and the multiplier *x* incorporates different processes depending on the life stage. All transitions (each arrow in Figure 1) occur simultaneously, each with a different value of *x* and *N*_state→_(*T*); *x* is derived as detailed below, while *N*_state→_(*T*) is simply the state‐dependent number of individuals as per last week's population state.

The transition that yields the youngest larval age class (arrow pointing left, Figure 1) has *x* = *C*_*D*_ *γS*(*T*): each adult female produces *C*_*D*_ eggs, of which a proportion of *γ* hatch (a value of *γ* < 1 penalizes offspring of hybrid) and thereafter survival *S*(*T*) ≤ 1 is applied before the larvae become age 1 (see ‘Density‐dependent survival’, below, for the value of *S*(*T*)).

We use *x* = *S*(*T*) for larvae transitioning from ages 1 to 5 weeks to the next age class. From 6 weeks onwards (*i* ≥ 6), larvae may become pending, with probability *S*(*T*)*m*_*i*_. We additionally start tracking the sex of larvae from the pending state onwards, thus *x* = 0.5 × *S*(*T*)*m*_*i*_ for the transition from an unsexed larva to a pending male, and the same value *x* = 0.5 × *S*(*T*)*m*_*i*_ applies to the transition from an unsexed larva to a pending female. The sequence of *m*_*i*_ values (*i* = 6, 7, …, 11) is designed to reflect that development takes six to eleven weeks, independent of the timing strain (Supporting Information the sequence of values reflecting biologically realistic *m*_*i*_ values). Surviving larvae that do not become pending remain non‐pending (and thus also unsexed), with 1 week added to their age (probability is *S*(*T*)(1 − *m*_*i*_)). To reduce the number of states that need to be tracked, we consider larvae unsexed if they have not yet transitioned to a pending state. In reality they are already male or female, but in the absence of evidence of any difference in developmental rates or mortalities, the model does not require this information. Note that the same individual cannot become pending and emerge in the same week; this reflects the necessary time to complete pupation.

The above lists all values of *x* that are applied every week to all larvae, regardless of their strain. There are additional arrows in Figure 1 for which *x* = 0 in weeks with no suitable emergence cue. The responses to these cues are strain‐specific. *T* is a suitable week number, indicative of moon phases, thus for emergence from any pending state,

(2) $$x=\left\{\begin{array}{c} S\left(T\right)\\ S\left(T\right)\\ 0 \end{array}\;\begin{array}{c} \text{if}\;T\;\mathrm{mod}\;4=0\;\text{and the strain is}\;E_{M1}\;\text{or}\;E_{F}\;\\ \text{if}\;T\;\mathrm{mod}\;4=2\;\text{and the strain is}\;E_{M2}\;\text{or}\;E_{F}\;\\ \text{otherwise} \end{array}\right..$$

Here, mod is the mathematical modulo operator. Note that the above dynamic assumptions automatically lead to strain‐dependence of the maximum duration of the pending state. *E*_F_ individuals never spend longer than 2 weeks in this state while waiting for the next emergence opportunity, while *E*_*M*1_ and *E*_*M*2_ individuals spend up to 4 weeks in the pending state (during which they may also grow, see Section 2.7, below).

### 2.5 Density‐dependent survival: Determining the value of *S*(*T*)

To implement the transitions, we need the current value of *S*(*T*), the weekly survival of larvae, which depends on resource availability at time *T*. As larvae deplete resources, we express *S*(*T*) directly as a function of the number of currently existing larvae *N*_L_(*T*).

We model resources in two ways. In the *single niche* scenario, all larvae consume a shared resource, and weekly survival is reduced at high densities:

(3) $$S\left(T\right)=S_{\max}e^{-\alpha N_{L}{\left(T\right)}^{2}}.$$

*S*_max_ gives survival under unlimited resources, *N*_L_(*T*) denotes the current total population of all larvae (including pending larvae *N*_♂P_ + *N*_♀P_) and *α* indicates the strength of density dependence: high *α* makes survival decline faster with larval population size. The quadratic term ensures that density dependence remains mild at low densities.

In the *niche shift* scenario, young and older larvae utilize Niche 1 and Niche 2, respectively, and the niche shift occurs at *w* weeks of age. The population size of young larvae using Niche 1, *N*_n1_, at time *T* is

(4a) $$N_{n1}\left(T\right)=\sum_{i=1}^{w-1}N_{Li}\left(T\right),$$

where *N*_L*i*_ denotes the population size of larvae of age *i*. The population size of older larvae feeding on Niche 2, *N*_n2_, also includes pending larvae:

(4b) $$N_{n2}\left(T\right)=\left(\sum_{i=w}^{11}N_{Li}\left(T\right)\right)+N_{♂P}\left(T\right)+N_{♀P}\left(T\right).$$

Dynamics are thereafter derived assuming that Equation (1) applies separately for the survival of younger and older larvae, with {*N*_n1_, *α*_n1_, *N*_n2_, *α*_n2_} replacing {*N*_L_, *α*} of the *single niche* scenario.

The {*α*_n1_,*α*_n2_} combination that yields the same population size as *α* is not uniquely defined, as the dynamics depend on relative strain frequencies, the hybridization regime and whether all females mate. Pragmatically, we chose {*α*_n1_,*α*_n2_} values that gave similar adult population sizes as *α* does in the single niche scenario when assuming no mate limitation and only one strain present (Figures 3a and 4a). As fewer old than young larvae are available to contribute to density dependence, an equivalent level of competition requires *α*_n1_ < *α*_n2_ and we focus on this setting in the niche shift scenarios.

### 2.6 Mating dynamics

If there are emerging larvae of at least one strain, the computation of the weekly dynamics is interrupted by a more detailed consideration of matings. In nature, emergence is spread out over several days (Kaiser et al., 2021) with non‐overlapping reproduction events. We therefore split the population of emerging adults (all *N*_→state_ where the state is an adult) to five mating pools, with 10%, 20%, 40%, 20% and 10% of adults emerging on days 1–5. We assume all 5 days contribute to the same (weekly) cohort of offspring when measured over the slower time scale relevant for larval development.

Within each of the 5 days of an adult emergence week, adults emerge and spread over a small geographic area and mate during a time window of a few hours. We use *t* to denote time flow at this scale.

We contrast two assumption sets to be able to separate the effects of pure ecological competition from those that also involve hybrid production with potentially asymmetric effects on the parental strains. In the *ecological competition* scenario, strains form separate mating pools, even if they co‐emerge temporally, preventing hybridization. In nature, this is possible if strains emerge on the same day but at starkly different times of the day (Kaiser et al., 2021). In the *random mating* scenario, all matings are permitted to occur, with no assortativeness during each mating window.

We use a standard mass action formulation (Berec & Boukal, 2004; Hutchinson & Waser, 2007; Kokko, 2024), where pairs form at a rate *bN*_♀_(*t*)*N*_♂S_(*t*), where *b* > 0 is a constant that denotes mate search efficiency, and *N*_♂S_ is the searching subset of males (males cannot search while already occupied with a mating). Encounters make females disappear from the pool of available (virgin) females, while for males, we assume that mating takes on average *τ* units of time, after which they resume searching. This leads to the dynamic equations

(5a) $$\frac{{\mathrm{dN}}_{♀}}{\mathrm{dt}}=-bN_{♀}\left(t\right)N_{♂S}\left(t\right),$$

(5b) $$\frac{dN_{♂S}}{\mathrm{dt}}=-bN_{♀}\left(t\right)N_{♂S}\left(t\right)+\frac{N_{♂o}\left(t\right)}{\tau },$$

(5c) $$\frac{dN_{♂o}}{\mathrm{dt}}=bN_{♀}\left(t\right)N_{♂S}\left(t\right)-\frac{N_{♂o}\left(t\right)}{\tau },$$

where *N*_♂O_(*t*) denotes the number of occupied males at time *t*.

We assume all females and males scheduled to emerge on a given day are present in the beginning of the mating window (*t* = 0), with none of the males in an occupied state (*N*_♂O_(0) = 0). We assume no protandry (no earlier emergence of males relative to females), which aligns with observations as well as being the evolutionary prediction when males cannot mate with many females before dying (Ekrem & Kokko, 2023). The dynamics ends at a set time *t*_end_, and the outcome of interest is the probability *p* that a female was mated, obtained by solving Equations ((5a), (5b), (5c)) numerically to find $N_{♀}\left(t_{\mathrm{end}}\right)$ and inserting its value to

(6) $$p=1-\frac{N_{♀}\left(t_{\mathrm{end}}\right)}{N_{♀}\left(0\right)}.$$

We use *τ* = 0.25 and *t*_end_ = 1, which allows 4 matings per male, but for finite values of *b*, males ‘waste’ some time searching for females and consequently achieve a lower number of matings.

We contrast our main findings with an option where any mate finding limitation is switched off: here we simply set *p* = 1 (all females mate) without explicit tracking of the mating dynamics.

### 2.7 Reproduction

Each fertilized female produces an egg clutch of size *C*_*D*_, where *D* is the time she spent in the pending stage (Figure 1). We consider different options for the relationship between *D* and *C*_*D*_, for example, *C*_*D*_ = 60, 90, 120, 150 for *D* = 1, 2, 3, 4 respectively, or a flat line that assumes no growth while pending.

Eggs hatch to become larvae of the youngest age class, forming a cohort that enters the weekly dynamics. All pure‐strain eggs enter this cohort, but only a proportion *γ* of hybrids do. The proportion of eggs in each genotype category follows Mendelian inheritance rules (without stochasticity) with mothers as well as sires chosen in proportion to their genotype frequency in the mating pool. The ecological competition scenario has no hybrids, thus offspring inherit the mother's genotype, this being identical to that of the father.

All adults die at the end of the mating window (at time *t*_end_).

### 2.8 One‐season and multi‐year dynamics

**FIGURE 2:**
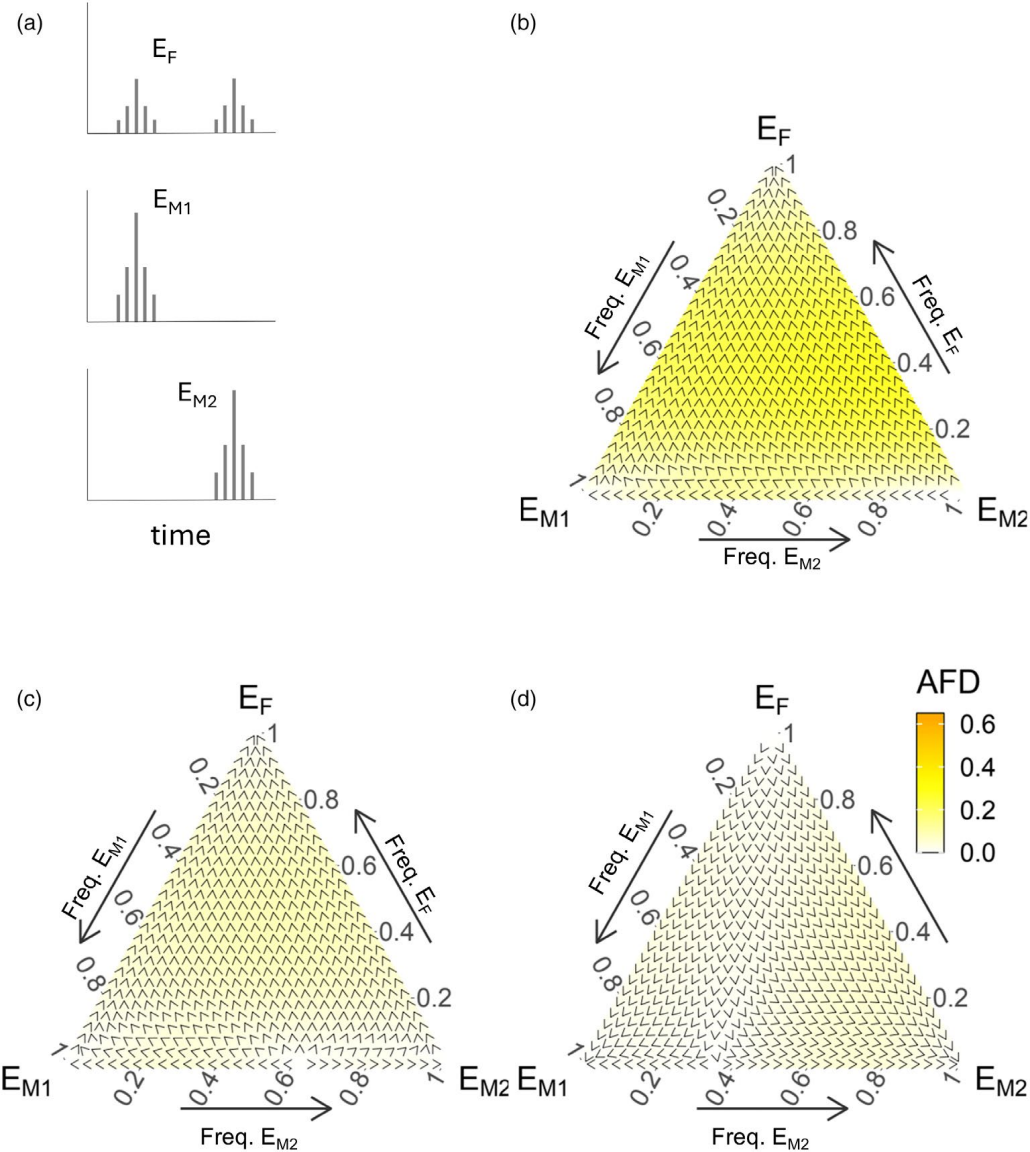
(a) The adult emergence of the fortnightly (*E*_F_), and monthly (*E*_*M*1_ and *E*_*M*2_) strains over 1 month. (b–d) Allele frequency dynamics for the case of ecological competition (no hybridization), a single niche for all larvae and no mate‐finding Allee effect (female mating probability *p* = 1). Small arrowheads indicate the direction of allele frequency change, colours the overall rate of evolutionary change (darker corresponds to faster change), quantified with Equation 7. Parameter values: *S*_max_ = 0.85, *α* = 10^−8^, *t*_end_ = 1. (b) In the absence of a growth‐survival tradeoff (*C*_*D*_ = 100 for all *D*), all cases lead to *E*_F_ outcompeting all others except if *E*_F_ is absent (along the *E*_*M*1_–*E*_*M*2_ axis), in which case the strain with the head start, *E*_*M*1_, wins. (c) A clear growth‐survival tradeoff, *C*_*D*_ = 60, 90, 120, 150 for *D* = 1, 2, 3, 4 respectively, only changes the result in that the *E*_*M*2_ strain can win if initially common and if only competing with *E*_*M*1_ (*E*_F_ absent). *E*_F_, if present, still outcompetes both monthly strains. (d) An even stronger growth‐survival tradeoff, *C*_*D*_ = 40, 80, 120, 160 for *D* = 1, 2, 3, 4, can make either *E*_*M*1_ or *E*_*M*2_ outcompete *E*_F_, with the outcome largely determined by initial abundances.

**FIGURE 3:**
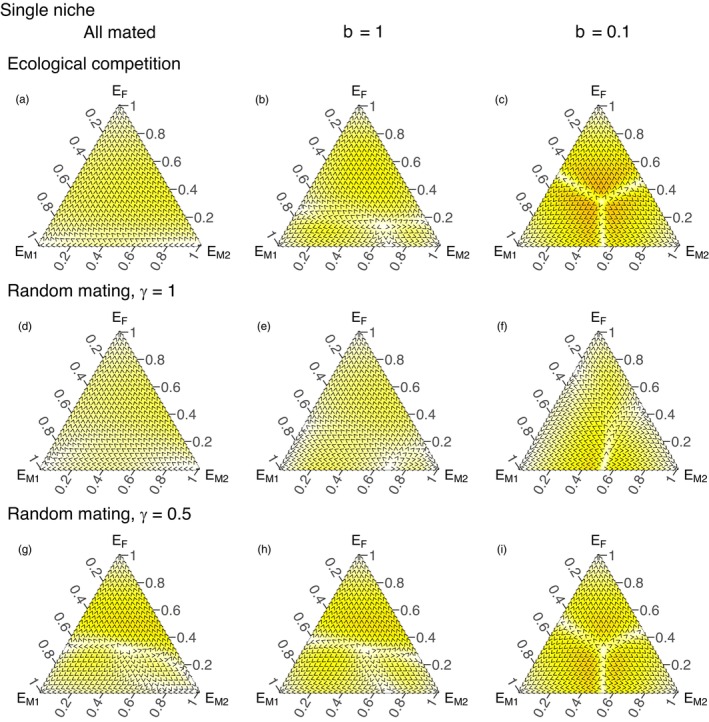
Allele frequency dynamics for the case of the single niche scenario with density dependence parameter *α* = 10^−8^, for the (a–c) ecological competition scenario, where *γ* is irrelevant, and the (d–i) random mating scenario, with hybrid viability (*γ*) as indicated. Columns refer to the strength of the mate‐finding Allee effect: (a, d, g), all females mate (*p* = 1, no Allee effect); (b, c, e, f, h, i), mate‐searching efficiency as indicated by the value of *b*, with lower values implying a stronger Allee effect as indicated. Other parameter values as in Figure 2b. The priority effect is at its strongest (basins of attraction are at their most symmetric) in (c) and (i).

**FIGURE 4:**
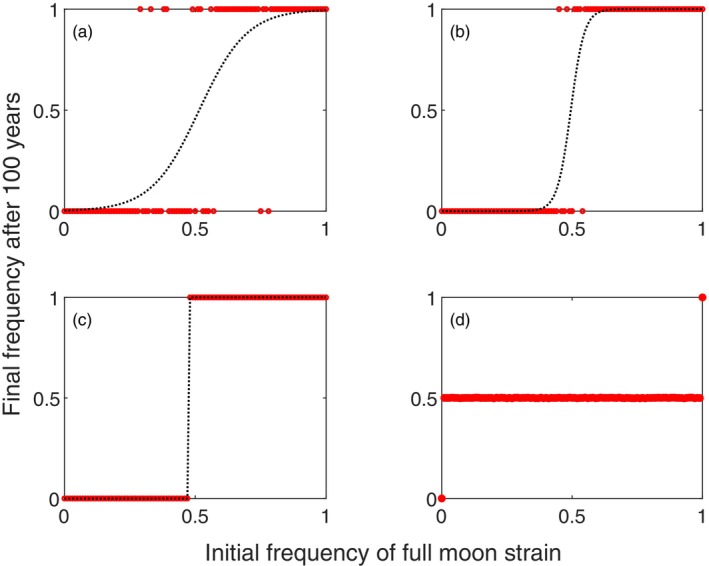
Results over 100 years in the multi‐year simulation, extending the horizontal axis results of figures (a) 3a, (b) 3b, (c) 3c and (d) 5a. Each red dot is a population that is initialized at a specific frequency (*x* axis) of full moon eggs, with the competitor strain (complementary frequency 1 − *x*) being the new moon strain; the fortnightly strain is absent in these examples. Only one strain prevails after 100 years in examples that feature a priority effect (a–c), and for these cases, we plot the predicted winning probability based on a logistic regression for the win of the full moon strain as a function of its initial frequency. In (d), where the single‐season dynamics (Figure 5a) predicted genotype shifts towards equal abundance, all simulations except those that were initialized with only one strain present (frequencies 0 or 1) yielded 100‐year dynamics that lead to coexistence, with each strain forming 50% of the population.

We initialized each run with 10,000 larvae of the youngest age class, comprising three possible homozygotes {*E*_F_ *E*_F_, *E*_*M*1_ *E*_*M*1_, *E*_*M*2_ *E*_*M*2_} covering the possible frequency space with equidistant intervals (sum of frequencies = 1). The end‐of‐season frequencies can be likewise reported as homozygote frequencies in the ecological coexistence scenario, but in the random mating scenario, there are also heterozygotes. For simplicity, we therefore report allele (not genotype) frequencies at the end of season. This makes it possible to use ternary plots (Figures 2, 3, 4; Figures S1, S2 and S4) for frequencies and their changes for all scenarios. Points along the ternary plot edges depict competition of two alleles in the absence of the third. The direction of arrows along the edges shows which of the two competitors is increasing in frequency. Points in the interior of the ternary plot have all three alleles (at least temporarily) present; here, the arrow shows the current shift of the abundances of the three competing alleles.

To summarize the total change that allele frequencies experience over one season, we adopt Berner's (2019) measure of genetic difference, allele frequency difference (AFD), which we for our purposes adapt to a temporal context,

(7) $$\mathrm{AFD}=\frac{1}{2}\left(\left|∆f_{E_{M1}}\right|+\left|∆f_{E_{M2}}\right|+\left|∆f_{E_{F}}\right|\right).$$

Each Δ*f* denotes allele frequency change in the egg pool between initialization and the last 4 weeks of the season (one lunar cycle). We consider the last 4 weeks, rather than the very last week, to avoid bias based on weekly fluctuations caused by the variable timing of egg production of the three strains.

In multi‐year simulations, we do not summarize results using AFD, as it summarizes the situation in a way that does not preserve all information needed to start the next year. Instead, we record the number of eggs of each genotype that the previous season produced, and normalize such that a total of 10,000 eggs survive the winter to begin the next season, with genotype frequencies identical to the production in the preceding season. Multi‐year simulations additionally need to take into account the physical fact that the start of the season is dictated by water temperatures, not the phase of the moon. The randomization procedure (see Life cycle, above) for the moon phases makes the year start at one of four options 1, 2, 3 or 4 for the first week, which breaks any correlation between the moon phase and the start of the season.

## 3 RESULTS

### 3.1 Difference in timing of reproduction creates priority effects, not coexistence

Our first example assumes no hybridization (only ecological competition between larvae), a single niche, and that all females mate (*p* = 1). We find no coexistence (Figure 2b,c). Instead, the presence or absence of a growth‐survival tradeoff, as well as its strength when present, determines the winning strain. If the tradeoff is absent, the fortnightly strain *E*_F_ benefits from reproducing at the first available opportunity, and it will win (Figure 2b, arrows pointing upwards)—unless it is initially absent, in which case the strain with the head start, *E*_*M*1_, wins instead (arrows pointing left along the *E*_*M*1_–*E*_*M*2_ axis, Figure 2b). Being able to reproduce at the earliest opportunity determines winning, as there is no reason to delay reproduction either to achieve synchrony—we here assumed no Allee effects—or to grow.

Incorporating a growth‐survival tradeoff (Figure 2c) changes the picture only modestly. *E*_F_ still wins if it is initially present, but in its absence, the head‐starting strain *E*_*M*1_ is no longer always the winner. Instead, *E*_*M*2_ wins if it is common to begin with (above 0.6). The initial advantage of reproducing early (the reason *E*_*M*1_ won in Figure 2b) now comes with a downside: *E*_*M*1_ is less fecund in the very beginning of the season than *E*_*M*2_; the latter has more time to grow as it waits for a suitable moon phase. Finally, if the growth‐survival tradeoff is very strong (Figure 2d), *E*_F_ is not maintained in the system. It has, on average, the shortest developmental duration, which is penalized when long growth improves fecundity. Also, the head‐start strain *E*_*M*1_ needs a marked initial advantage (0.6 or higher) for it to be the winner of an *E*_*M*1_–*E*_*M*2_ competition (read along the horizontal axis of Figure 2d: *E*_*M*2_ must to be initialized below 0.4 for *E*_*M*1_ to win). The monthly strain that is slow to start (and hence emerges at a larger size in the beginning of the season, *E*_*M*2_) has the largest basin of attraction.

Summing up results thus far, different temporal niches do not automatically aid coexistence. Even though larvae of a specific strain cease to be competitors for larval resources at emergence (which in principle boosts the survival of the remaining larvae of the competitor strain), the release from competition is only temporary. The emerged strain nearly immediately adds new competitors via eggs that then hatch to be larvae. When most of life is spent in the larval stage, non‐overlapping adult temporal niches do not make larval niches non‐overlapping in time. Accordingly, Figure 2 produced no example where two or more strains coexist.

### 3.2 Priority effects are strongest when Allee effects combine with few (viable) or no hybrids

Above we assumed that mate finding is unproblematic, and that all matings occur within a strain. If reality deviates from these assumptions, a strain that currently occurs at higher frequency should benefit from improved availability of potential mates. This should strengthen any priority effects (sensu Fukami, 2015), where initial abundance helps a strain to outcompete others.

Under varying Allee effects and prospects for hybridization (Figure 3), we find that the strongest priority effects are found when the Allee effect is strong (low *b*, Figure 3c,i) and when hybridization is either absent (Figure 3c) or can occur but leads to maladapted offspring (Figure 3i). The more symmetrical the pattern, with fast movement away from the center of our heatmaps, the stronger the priority effects we observe. A strong Allee effect makes strain‐specific properties lose significance as a determinant of the outcome, and the winner is instead determined by initial abundance. High initial abundance reduces the risk of remaining unmated, and positive frequency dependence amplifies abundance differences over time.

If hybridization is unproblematic, the Allee effect loses some of its ability to create priority effects (Figure 3d–f): the priority effect caused by low mate searching efficiency is mitigated by allowing strains to hybridize without a cost (Figure 3e,f). But when hybrids perform poorly, rare strains suffer from taking disproportionately often part in hybrid production, and strong priority effects reappear (Figure 3i).

In multi‐year simulations, priority effects, unsurprisingly, yield one winner (Figure 4a–c). However, there is an interesting twist that relates to the fact that it made sense for us to use the nomenclature *E*_*M*1_ for earlier starting monthly strains and *E*_*M*2_ for the later starting one, as either the full or the new moon strain could take the early (the moon not being a determinant of water temperatures, which in turn dictate the season start). In multi‐year simulations, one strain is not committed to ‘being’ *E*_*M*1_ every year; the moon phase dictates which of the two monthly strains enjoys a head start, and this means a considerable stochasticity to the outcome when both strains initially occur at moderately high frequencies (Figure 4a,b). An exception occurs when the single‐season dynamics very strongly pushes the initially common strain to high abundances (Figure 4c, which places Figure 3c in a multi‐season setting). As a whole, priority effects yield just one surviving strain, but predicting which one it will be is not easy for large parts of the parameter range.

In summary, while the strength of the priority effect varies somewhat between the investigated scenarios (Allee effect, whether hybridization is allowed, and whether hybrids are assumed less fit), we so far have not reported a finding with negative frequency dependence that would protect a polymorphism with two or more strains. Additional investigations with growth‐survival tradeoffs interacting with Allee effects and hybridization do not introduce coexistence either (Figure S1). Instead, this tradeoff merely improves the winning chances of ‘more patient’ strains (monthly emergers) over ‘impatient’ ones (fortnightly), similarly to the logic of Figure 2.

### 3.3 Ontogenetic niche shifts can create negative frequency dependence when interacting with reproductive timing

Thus far, our examples did not include ontogenetic niche shifts. The long and variable duration of the larval stage makes complete competitive separation of *C. marinus* strains unrealistic. Still, incorporating niche shifts might make our case replicate a well known feature of non‐temporally defined niches: complete separation of larval niches should not be necessary for coexistence (Leimar et al., 2013).

**FIGURE 5:**
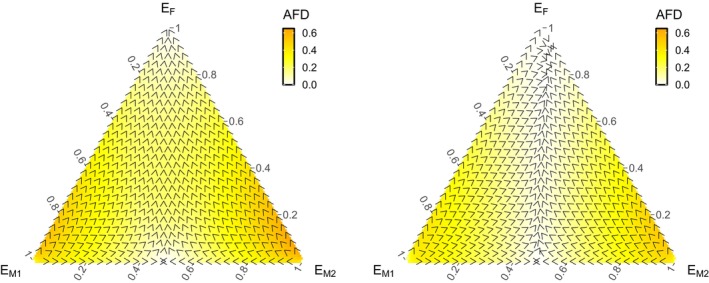
A suitably timed ontogenetic niche shift can aid coexistence. The figure uses Figure 2b,c's logic: The left triangle features no growth‐survival tradeoff (*C*_*D*_ = 100 for all *D*) while the right triangle uses a clear growth‐survival tradeoff with *C*_*D*_ = 60, 90, 120, 150 for *D* = 1 to 4; but here, the niche shift scenario is implemented with an ontogenetic niche shift that occurs at age of 2 weeks (*w* = 2), with density dependence parameters *α*_*n*1_ = 10^−8^ and *α*_*n*2_ = 2.6 × 10^−8^ indicating stronger density dependence in Niche 2. All other parameters are as in Figure 2.

Consider a population consisting of two strains *E*_*M*1_ and *E*_*M*2_ in the ontogenetic niche shift scenario (along the bottom edge of the ternary plot, Figure 5a). Each cohort is produced by a single strain as *E*_*M*1_ and *E*_*M*2_ choose different moon phases to reproduce, but cohorts will mix in a pool of different‐aged larval competitors. A niche shift at the age of 2 weeks (Figure 5a) helps separate the cohorts: at any given time, the niche for young larvae is occupied by either *E*_*M*1_ or *E*_*M*2_, but not both (for a visualization, see Figure S8). In Niche 2, *E*_*M*1_ and *E*_*M*2_ larvae mix, alternating between being over‐ and underrepresented (again, Figure S8 provides intuition). Thus, each of the monthly strains competes solely with its own cohort while in Niche 1 and predominantly with its own cohort while in Niche 2. A niche shift at 2 weeks therefore concentrates larval competition to mostly occur within a strain, promoting stable coexistence. The multi‐year version of this simulation confirms that strains then stabilize at 50% abundance each (Figure 4d).

However, this reasoning loses validity when competition occurs between a monthly (either *E*_*M*1_ or *E*_*M*2_) and a fortnightly (*E*_F_) strain. Now cohorts alternate between *E*_F_ on its own and together with its monthly competitor. *E*_F_ cohorts can enjoy protection from competition some of the time: half of all the tides that it uses for reproduction have no monthly competitor adding same‐age larvae as competitors. The monthly strain, however, enjoys no situation where it can avoid competition from *E*_F_, as *E*_F_ uses both the full and the new moon for emergence. The overall ‘impatience’ of *E*_F_ is rewarded under the ontogenetic niche shift scenario (Figure 5a), just like in the single niche scenario (Figure 2b). The situation is a little different if we also introduce a significant growth‐survival tradeoff, which penalizes the fortnightly *E*_F_ strain (Figure 5b). Now the maximally patient strain (*E*_*M*2_) can coexist with *E*_F_, if *E*_*M*1_ is absent.

Finally, consider a population that is initialized with all three strains (interior regions of Figure 5). New cohorts alternate between two mixes of strains: for 2 weeks, competition among young larvae involves *E*_F_ and *E*_*M*1_, switching to *E*_F_ and *E*_*M*2_ for the following 2 weeks. A niche shift provides no release from interstrain competition when all strains are frequent, and coexistence among all three strains is not helped by ontogenetic niche shifts.

Our lessons are generalizable to other parameter settings, including different assumptions about genetic dominance, adding Allee effects on top of the survival‐growth trade‐off (it strengthens priority effects in that context too), and variations in the age at which a niche shift (if present) occurs; see Supporting Information for details. The last of these factors generalizes in the sense that the outcome is rather sensitive to the exact details of the ontogenetic niche shift: suitably timed ones promote coexistence, unsuitably timed ones do not (Supporting Information).

## 4 DISCUSSION

Temporal niche partitioning is suspected to aid the maintenance of diversity (theory: Chesson, 2000; Gao et al., 2020; Mougi, 2020; empirical examples: Albrecht & Gotelli, 2001; Kelly & Bowler, 2005; Hamelin et al., 2016; Menges et al., 2024; within‐species polymorphism, Graipel et al., 2014). There are, however, both old (Case & Gilpin, 1974) and new (Stump & Vasseur, 2023; van Doorn et al., 2025) warnings against straightforwardly assuming that as soon as temporal differentiation has been found, coexistence has been explained. In the case of van Doorn et al. (2025), for example, simplistic expectations that phenotype matching should make divergence and its maintenance easy (an individual can only mate with those that co‐occur in time) fall apart when examining timing traits and their evolution in detail. Our study adds to the new literature by introducing another example where stark temporal differentiation of mating niches requires very specific circumstances for it to promote the coexistence of strains.

For *Clunio*, purely temporally defined mating niches typically result in a priority effect (Zou & Rudolf, 2023). During a single season of multiple generations, this promotes the abundance of a strain that was common to begin with; over multiple years, we predict a complete loss of diversity with one winner strain left. The effects operating in our system are, in the classification of Fukami (2015), examples of an inhibitory priority effect, where a strain that has become sufficiently frequent enjoys elevated fitness and outcompetes its competitors via positive frequency dependence.

Priority effects are an interesting phenomenon, but not an explanation for coexistence. *Clunio marinus* features multiple types of frequency dependence. Some are positive—promoting priority effects—while ontogenetic niche shifts are at least potentially negative. Negative frequency dependence can in principle promote polymorphisms, but when multiple effects interact, the outcome is not automatically coexistence‐promoting (for a conceptually similar result in insects with freshwater larvae metamorphosing into flying adults see Gómez‐Llano et al., 2023).

Examining the interactions between types of frequency dependence requires taking into account several factors in *C. marinus*. Adults mate but do not feed, and have no time to waste as adult lifespan is very short. The result is an Allee effect that disproportionately harms a currently rare morph. Hybridization has similar effects whenever hybrids are less fit than pure‐strain individuals: a rare strain is penalized because it is less easy for a rare strain to produce offspring without hybridizing. These factors all imply positive frequency dependence and thus priority effects (Ke & Letten, 2018; Zou & Rudolf, 2023). We can thus extend the message of Stump and Vasseur (2023), who warned against hasty conclusions that temporal niches, whenever they exist, are the causality behind coexistence. Our extension states that it appears worthwhile to study the alternative outcome of a priority effect as a likely end effect.

Still, not all hope is lost for temporal niches to play some role in creating negative frequency dependence for *Clunio* at the larval stage. Larvae might compete most strongly with others of the same strain, as they are placed in the competitive pool nearly simultaneously. Here we showed that the niches can become distinct enough competitive pools if there is a suitably timed ontogenetic niche shift. Even if larvae of a specific age sometimes compete with other strains (e.g. in one of the two temporal niches we modelled for the ontogeny), an ontogenetic niche shift can help intensify competition to occur mostly within a strain, without the need to assume different niches for different competitors (as in Schellekens et al., 2010). Empirically, we lack data for this aspect of *Clunio* biology, while theoretically, our present study showed that the timing of the niche shift has to be somewhat fortuitous for this to function well. At best, we can explain sympatric coexistence of two out of the three potential strains, but never three (this particular result is in line with current data, Kaiser et al., 2021). As a whole, our study encourages complementing research on temporal niches with priority effects (Zou & Rudolf, 2023) as well as factors outside temporal niches that might create species‐ or strain‐specific niche use (Ekrem et al., 2025; Stump & Vasseur, 2023).

## AUTHOR CONTRIBUTIONS

Runa K. Ekrem, Hanna Kokko and Tobias S. Kaiser conceived the idea. All authors contributed to designing and analysing the model. Runa K. Ekrem wrote a first draft of the manuscript. All authors contributed substantially to the drafts and gave final approval for publication.

## CONFLICT OF INTEREST STATEMENT

The authors declare no conflicts of interest.

## STATEMENT ON INCLUSION

This work is a modelling study with no primary data, and in that sense, there was no location for the data collection. The data that forms the scientific background originates mainly from European coasts and has already been published (we cite all relevant papers). We have striven to cite relevant papers from other systems from a wide variety of geographic regions (both with respect to species and the researchers studying them).

## Supporting information


**Appendix S1.** Table of variables and their meanings, computation of the daily emergence probabilities, and additional figures providing a robustness check.

## Data Availability

This is a modelling study with no original data. The code that produces the figures is available at https://zenodo.org/records/15519113.
