# Supplementary material for: Temporal niche differentiation often leads to priority effects rather than coexistence: Lessons from a marine midge
Source: J Anim Ecol. 2025 Jul 20;94(10):1935–47. doi: 10.1111/1365-2656.70094 (PMC12484382; doi:10.1111/1365-2656.70094)
Supplement: Supplementary file 1 — Appendix S1. Table of variables and their meanings, computation of the daily emergence probabilities, and additional figures providing a robustness check. [file JANE-94-1935-s001.docx]

# Supplementary material

## Table 1. Notation.

E_M1_, E_M2_, E_F_ Labels for the two monthly strains and the fortnightly strain, respectively

α Strength of density dependence in the single niche scenario

α_n1_, α_n2_  Strength of density dependence in niche 1 and 2, respectively, in the ontogenetic niche shift scenario

*b* Mate searching efficiency

γ Relative fitness of hybrids (γ < 1 indicates lower fitness than non-hybrids)

*C_D_* Clutch size of females that spent *D* weeks in the pending stage

*D* The number of weeks that a larva has spent so far in the pending stage

*f* Allele frequency (subscripts used to label the allele in question)

*N* Population size, with subscripts indicating restricting the meaning to a relevant stage (e.g. ♂S as a subscript implies the population size refers to searching males; see text for details for each subscript)

*p* Probability that an emerged female mated by the time the mating window is over

*T* Time, where integers refer to weeks

*t* Time, where continuous values refer to the time within one low tide

*t*_max_ Duration of the mating window during one low tide

τ Duration of one mating

*x* Transition probability between two different stages: individuals of a previous stage contribute *x* individuals of the subsequent stage to the population measured one week later

*S*(*T*) Weekly survival at week *T*

*S*_max_ Maximum survival (baseline which then is reduced through density dependence)

*m_i_* Probability that a larva of age *i* becomes pending (conditional on it surviving)

## Computation of the *m_i_* values

The sequence of *m_i_* values should yield uniformly distributed development time (ignoring mortality), such that in the absence of mortality 1/6th of the total initial population transitions to *pending* every week from week 6 to 11.

For week 6, the correct proportion is clearly *m_i_* = $\frac{1}{6}$. For week 7, it is no longer 1/6, since a proportion of $1-\frac{1}{6}$ = $\frac{5}{6}$ of individuals are left to be potential emergers, and we need to find a value of *m_i_* such that $\frac{5}{6}$ *m_i_* = $\frac{1}{6}$. The solution of this simple equation is *m_i_* = $\frac{1}{5}$. An analogous calculation for the remaining weeks shows that the sequence of *m_i_* values to be used is 1/6, 1/5, 1/4, 1/3, 1/2, 1 for *i* = 6, 7, 8, 9, 10, 11. The last value, *m*_11_ = 1, implies that everyone still left will emerge at the final chance to do so, and this is, as intended, 1/6th of the original population since $1-\frac{1}{6}-\frac{5}{6}\cdot\frac{1}{5}-\frac{4}{6}\cdot\frac{1}{4}-\frac{3}{6}\cdot\frac{1}{3}-\frac{2}{6}\cdot\frac{1}{2}=1-5\cdot\frac{1}{6}=\frac{1}{6}$.

In the modelled dynamics, mortality will reduce the size of the later age classes, thus the distribution of realized emergence times will be somewhat front-heavy.

# Robustness of results

Our lessons appear generalizable to other parameter settings than those discussed in the main text. Introducing Allee effects on top of the growth-survival tradeoff (Fig. S1) strengthens priority effects, making coexistence harder. This is easiest to see by examining the horizontal axis of each triangle in Fig. S1: when all females mate (Fig. S1A,D,G), the system predicts coexistence of two monthly strains even if one of them is rare to begin with. When Allee effects are strong, coexistence of the two is only possible if their initial abundances are not too different. If one is much more common than the other, the priority effect makes it win (arrows pointing towards the corners in Fig. S1C,F,I).

While a monthly strain can coexist at low abundance with a more frequently emerging strain (abundant E_F_ and some E_M2_ in Fig. 5B and S2A), such cases are fragile. They disappear when Allee effects operate or when the currently rare strain is disadvantaged through hybridization (all other panels in Fig. S2 except S2A). Both effects reduce the success of the rare strain and thus act against a protected polymorphism. Removing the growth-survival tradeoff (present in Fig. S3 but absent in Fig. S2) further penalizes monthly emerging rare strains. Some individuals of monthly emergers reproduce at a relatively advanced age and if their fecundity is no higher than that of their younger competitors, a potential competitive advantage is lost. The region of initial frequencies from which the monthly strains win is consequently smaller in Fig. S2B-C than in Fig. S3B-C. In Fig. S2B the monthly emerging strains only win if they start off at high frequencies (close to the EM1-EM2 boundary), whereas Fig. S3B has a clearly larger set of initial frequencies leading to monthly emergence winning.

We also explored ontogenetic niche shifts that occurred at later ages (Fig. S4-S7). Results for a shift at four weeks strongly resemble those of the *single niche* scenario (Fig. S4-S5), while a shift at 6 weeks produces results similar to a shift at two weeks (Fig. S6-S7). This is explicable: An ontogenetic niche shift after an entire lunar cycle has passed makes an older larval cohort stay in niche 1 long enough to compete substantially with a younger cohort of a different strain. If the shift is delayed further, to 6 weeks, an old larva will experience an influx of numerous young competitors of its own strain (young larvae are more numerous, as mortality has not yet thinned them) before being released from competition in niche 2; this latter niche is now less important as individuals spend less time in it when the niche shift occurs late in the development of larvae.

Changing dominance assumptions from E_M1_ and E_M2_ being dominant to E_F_ being dominant has minor effects on the outcome. For brevity’s sake we summarize these results verbally only: Making E_F_ dominant gives E_F_ some benefit under the random mating scenario (*γ* = 1) under low mate searching efficiency (*b*). All our qualitative results listed above are robust to this change.


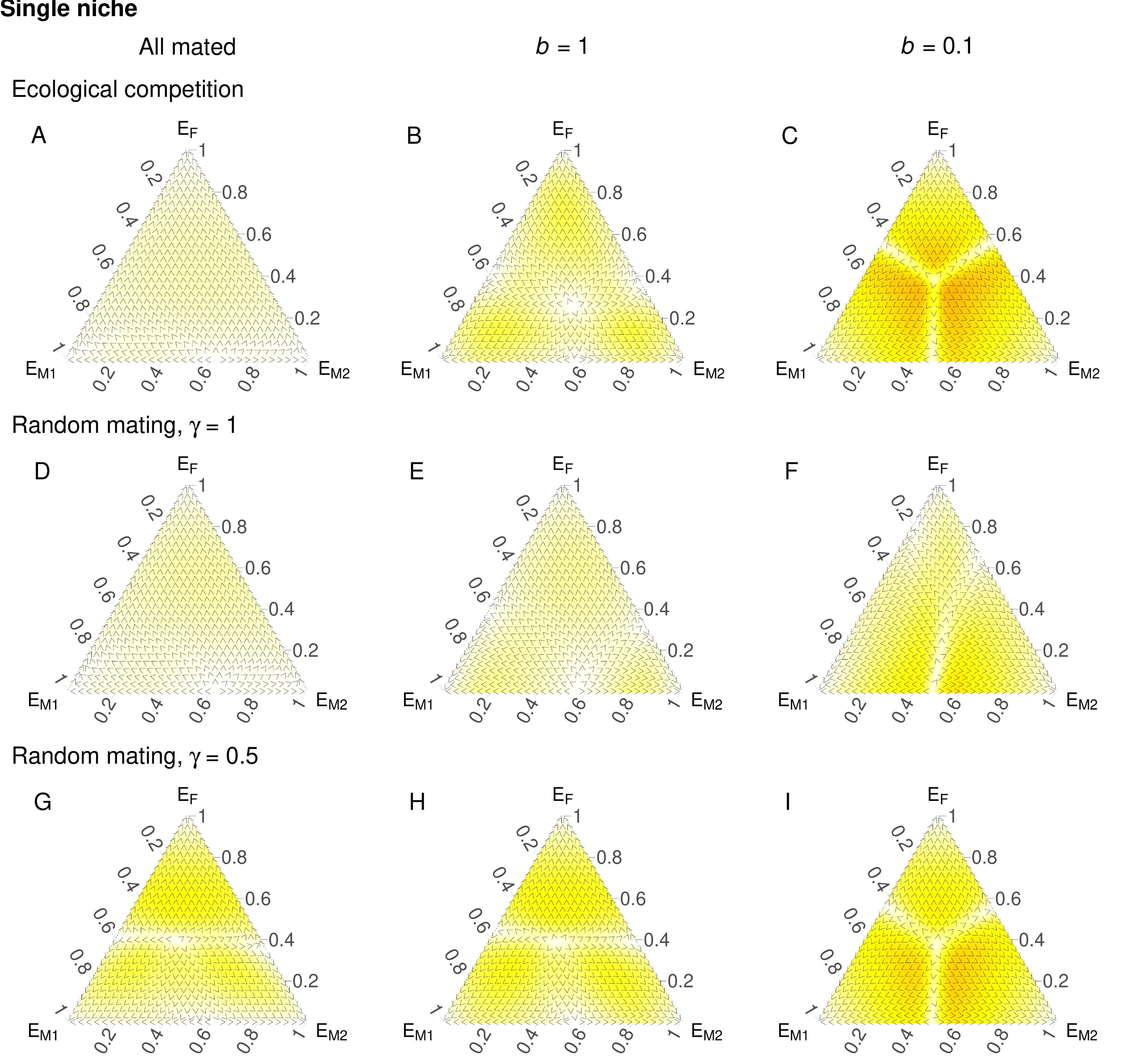


**Figure S1:** Like Figure 3**,** but with a growth-survival tradeoff, using *C_D_* = 60, 90, 120, 150 for *D* = 1, 2, 3, 4, respectively.


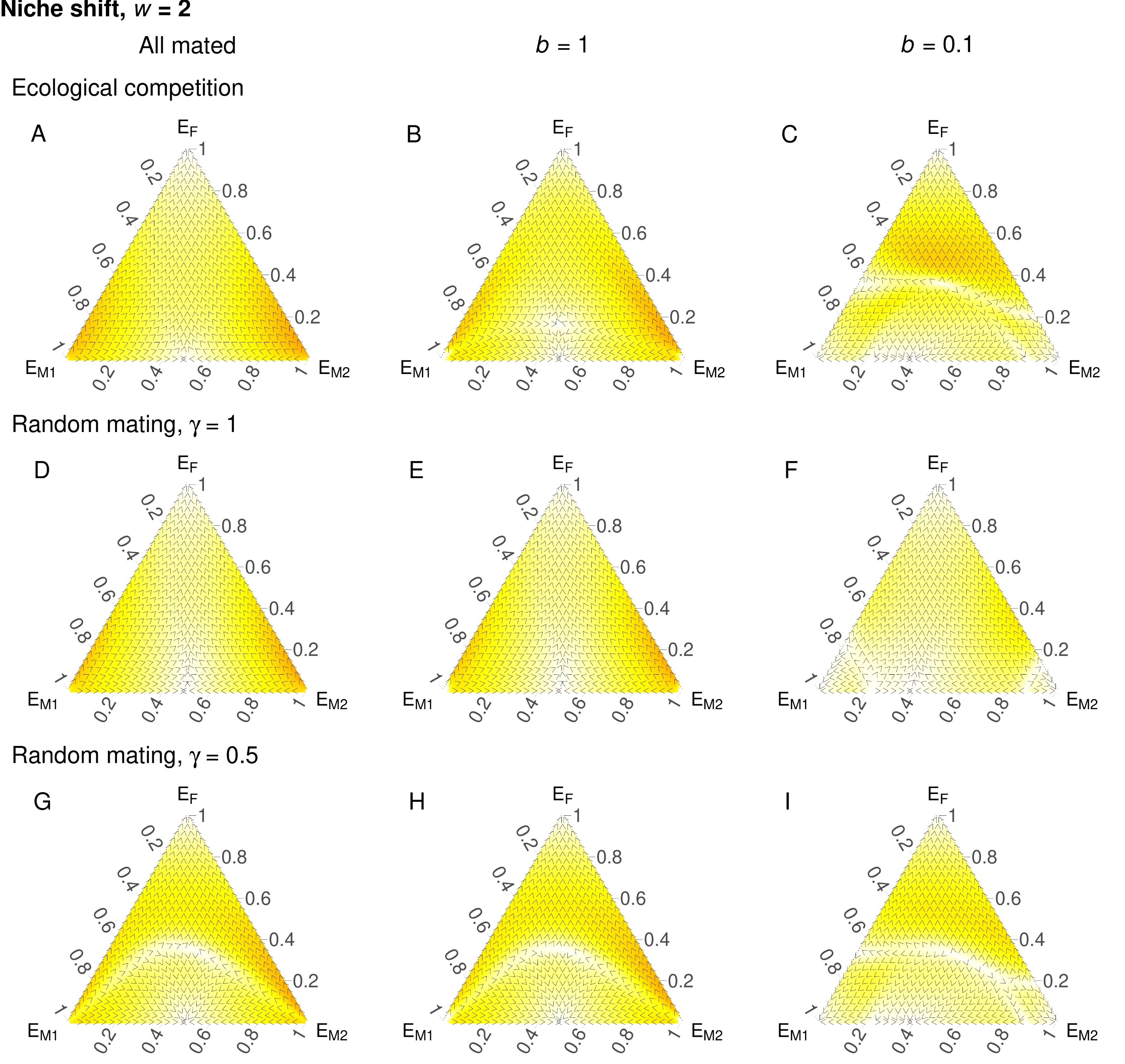


**Figure S2.** Like Fig. 5 (the same values for parameters including a niche shift at 2 weeks), but without a growth-survival tradeoff throughout the examples, and with varying other assumptions related to the mating dynamics as indicated (mating dynamics assumption variation between the triangles follows the logic of Figure 3).


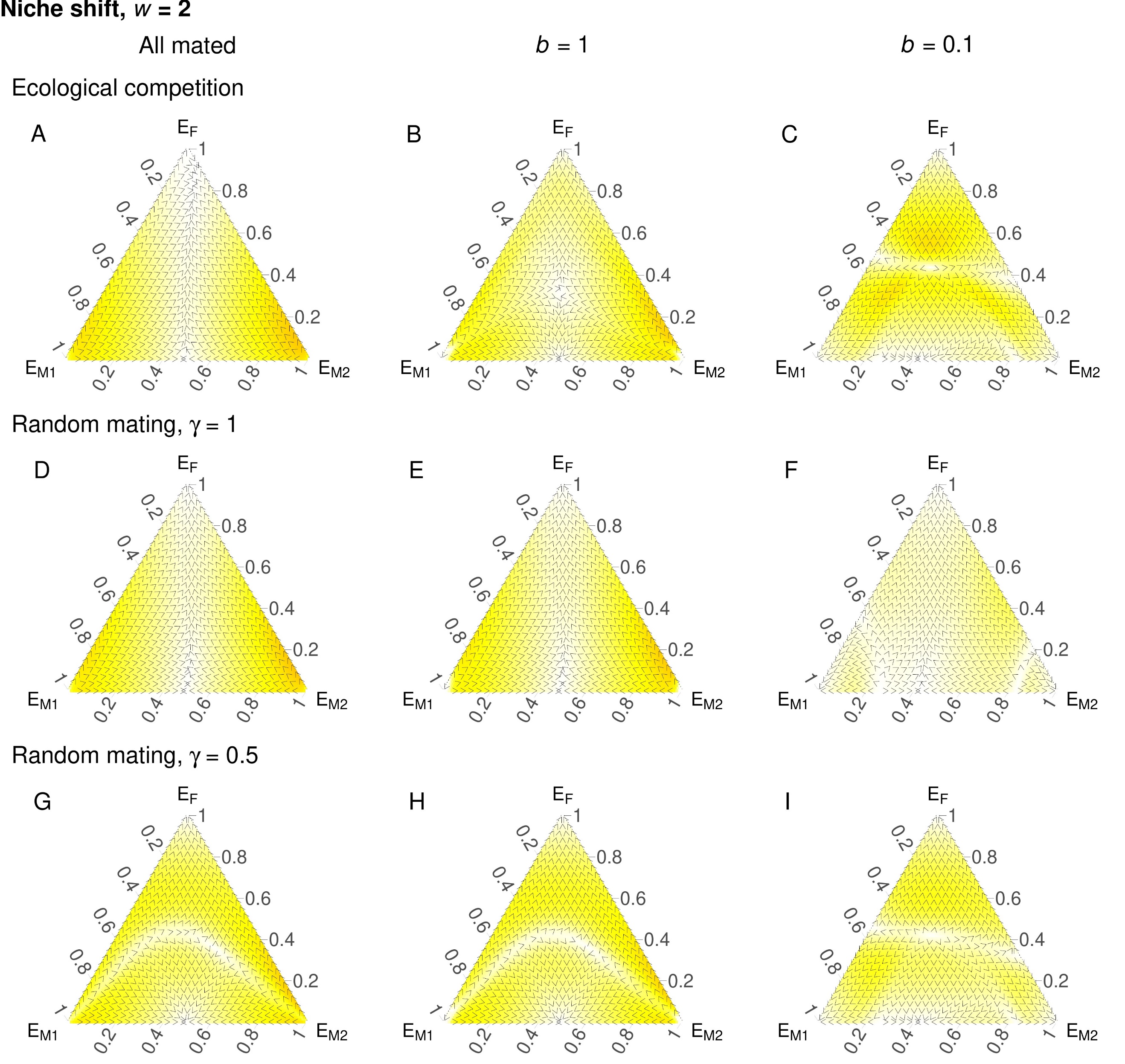


**Figure S3:** Like Figure S2, but with a growth-survival tradeoff, using *C_D_* = 60, 90, 120, 150 for *D* = 1, 2, 3, 4, respectively.


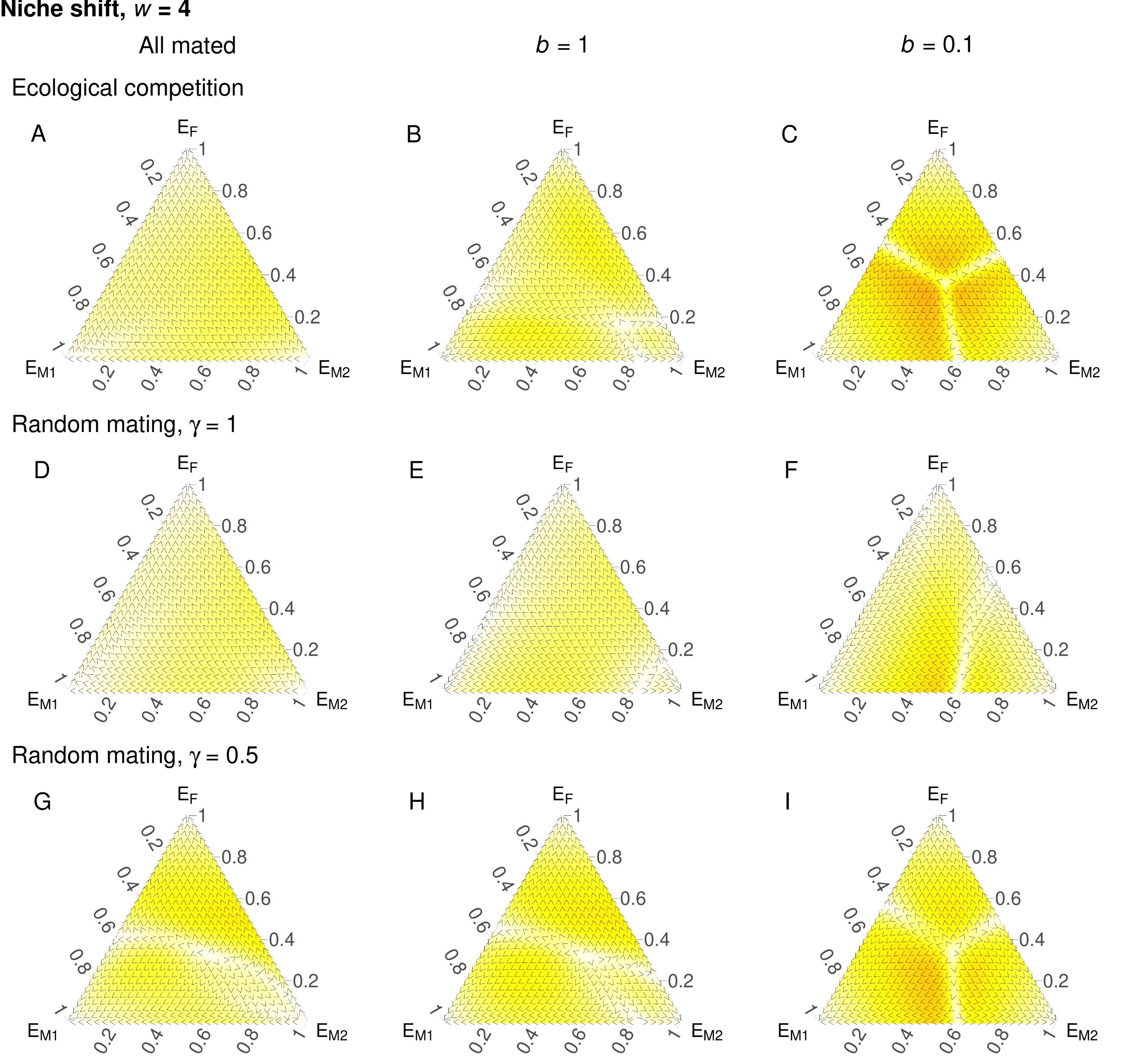


**Figure S4:** Like Figure 3 (no growth-survival trade-off), but with an ontogenetic niche shift that occurs at the age of 4 weeks (α_n1_ = 10^–8^ , α_n2_ = 1.96×10^–7^).


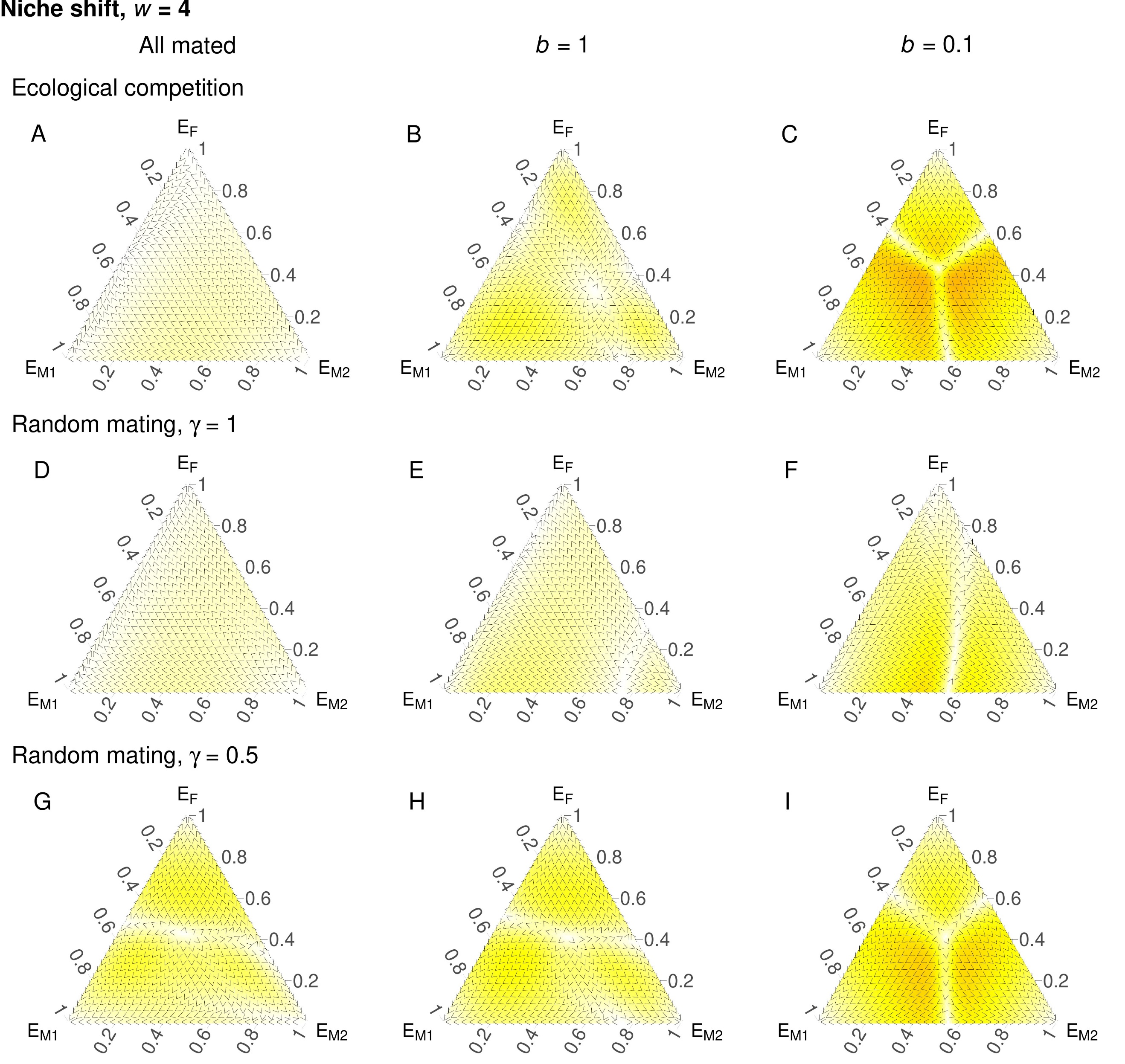


**Figure S5:** Like Figure S4**,** but with a growth-survival tradeoff, using *C_D_* = 60, 90, 120, 150 for *D* = 1, 2, 3, 4, respectively.


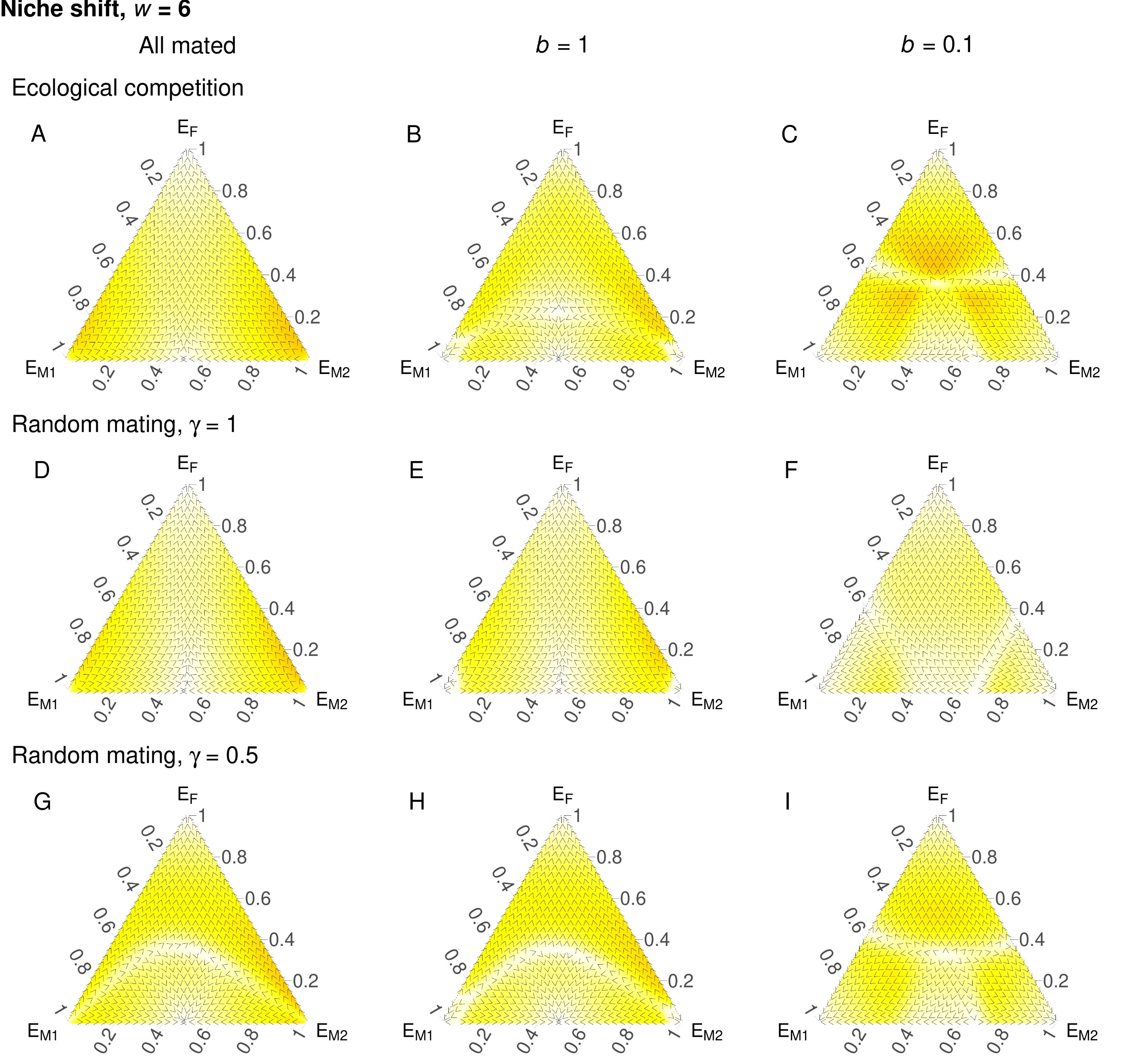


**Figure S6:** Like Figure 3 (no growth-survival trade-off), but with an ontogenetic niche shift that occurs at age 6 weeks (α_n1_ = 10^–8^ , α_n2_ = 10^–6^).


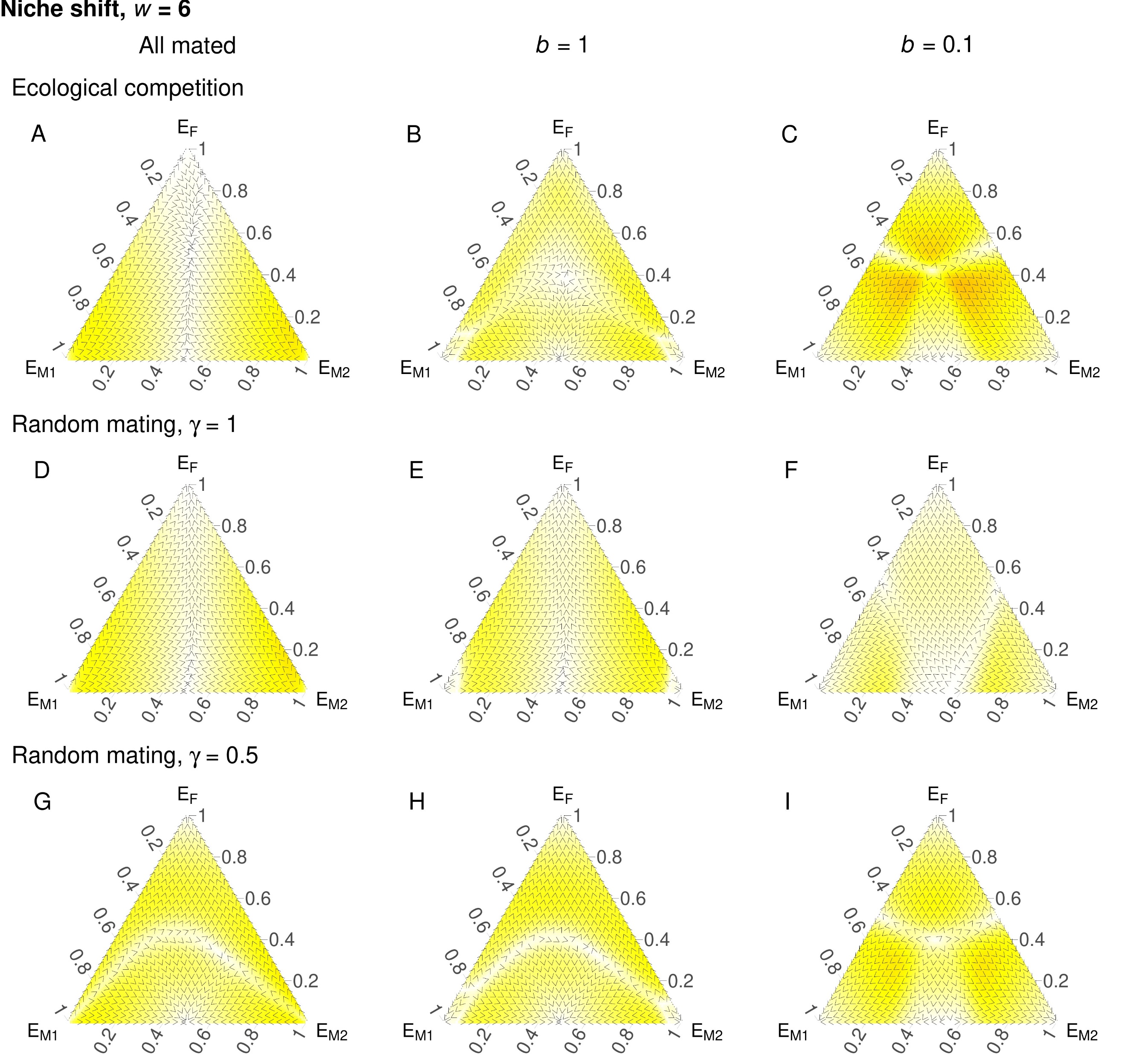


**Figure S7:** Like Figure S6**,** but with a growth-survival tradeoff, using *C_D_* = 60, 90, 120, 150 for *D* = 1, 2, 3, 4, respectively.


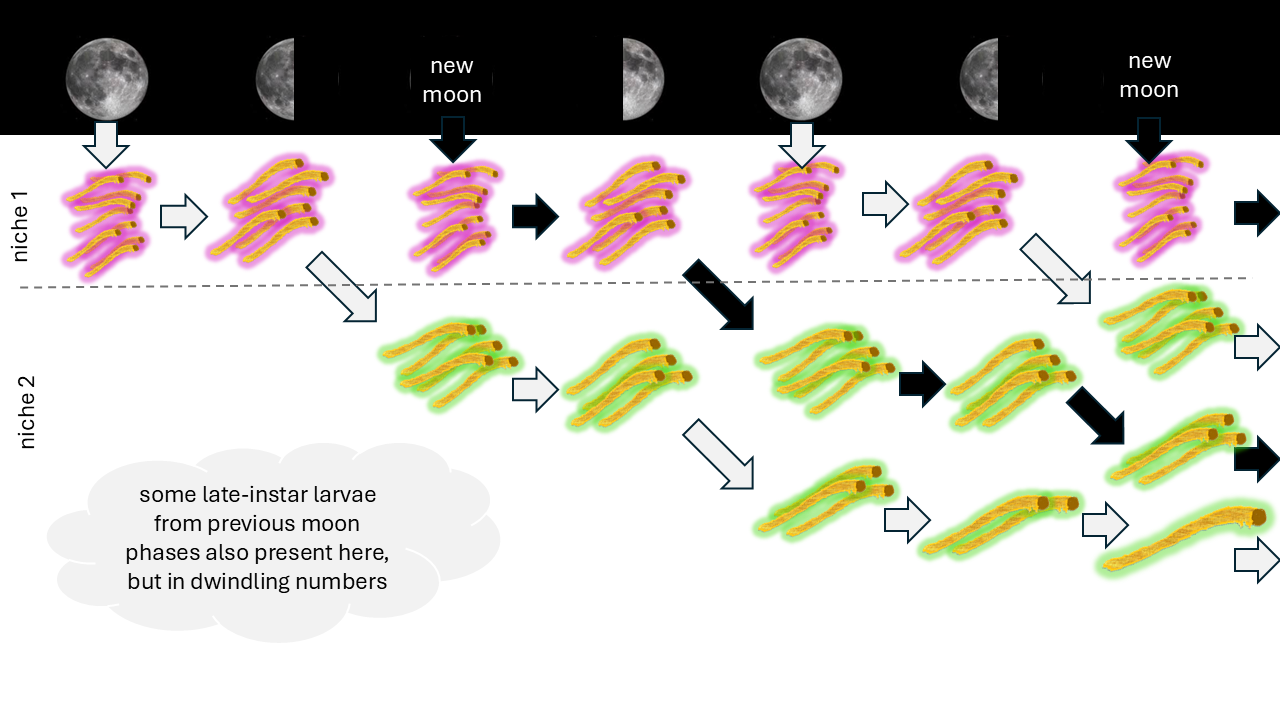


**Figure S8:** A conceptual visualization of the process that leads to the effects of a suitably timed ontogenetic niche shift. Larval growth is depicted with increasing sized larvae while mortality is indicated with fewer larvae present in older age classes, with full moon strains following the route of pale grey arrows and new moon strain the route of black arrows. Larvae begin their lives in niche 1 (pink resource) and transition, in this example after 2 weeks, to niche 2 (green resource). In niche 1, there is only one strain present at a time when the niche shift is at 2 weeks. In niche 2, abundances fluctuate: in the present example, the last exemplified week has more full moon than new moon strain individuals present, while the week before the abundances were biased in the opposite direction. The text bubble reminds the reader that the numbers of larvae are a conceptual aid, not a numerically accurate representation; a complete picture would also feature older larvae present from earlier moon phases that added larvae to the pool of competitors, but these are present in dwindling numbers as they begin emerging and exiting the water, and mortality also has had more time to thin their numbers than in the depicted cohorts.
